# Editorial: Emerging swine viruses, Volume II

**DOI:** 10.3389/fvets.2022.1065549

**Published:** 2022-11-02

**Authors:** Daniela S. Rajao, Carlos Juan Perfumo, Daniel Roberto Perez, Enric Mateu

**Affiliations:** ^1^Department of Population Health, University of Georgia, Athens, GA, United States; ^2^Faculty of Veterinary Sciences, La Plata National University, La Plata, Argentina; ^3^Department of Animal Health and Anatomy, Universitat Autònoma de Barcelona, Barcelona, Spain

**Keywords:** swine, virus, emerging, outbreaks, novel

The continuous growth of the human population and the need for resources has led to expanding encroachment into wildlife habitats (1). Increased interaction among humans, livestock, and wild animals can lead to spillover and spread of pathogens, particularly viruses, from wildlife reservoir species. The past few years have shown the risk of these increased interactions to the global population. The spread of the novel severe acute respiratory syndrome coronavirus 2 (SARS-CoV-2) resulted in a global pandemic (COVID-19), leading to more than 600 million cases and 6 million deaths worldwide, in addition to devastating social and economic consequences (2). Coincidently, another pandemic caused by African swine fever virus (ASFV) arose in domestic pigs in China just prior to the COVID-19 pandemic, reaching many other Asian and European countries. It is estimated that the outbreak resulted in the loss of about 30% of the Chinese pig population, leading to devastating consequences to the overall global swine industry (3, 4).

To meet the growing demand for meat and meat products, swine production has followed a similar growth trend (5). Following the COVID-19 pandemic, supply chain disruptions and increased meat prices are expected to reduce pork consumption and result in a slower growth than previous years. In countries with high swine production, production growth in the past 4 decades was accompanied by significant structural changes with a rapid shift to fewer and larger operations (5). These large production systems are characterized by tens of thousands susceptible animals concentrated in small areas. Current swine production systems involve continuous introduction and movement of pigs, often housing animals of different ages and immune status in the same facilities, providing the ideal environment for the introduction and spread of pathogens.

Next generation genome sequencing techniques are becoming routinely used in virus diagnostics, contributing to a better understanding of virus evolution and virus discovery (6). Metagenomics and transcriptomics technologies allow for agnostic approaches that lead to detection and full genome characterization of potential pathogens in a sample, without the need to know about the organisms being investigated or to isolate and grow viruses in a laboratory. These technologies contributed to the discovery of novel organisms such as giant viruses present in environmental samples, characterization of human, animal, and environmental viromes, in addition to the detection of novel emerging and reemerging viral pathogens (6, 7).

Novel viruses of pigs have been detected in the last 3 to 4 decades at a higher speed than ever before. Among the viruses that resulted in the highest economic losses are re-emerging porcine reproductive and respiratory syndrome (PRRS), porcine circovirus type 2 (PCV2), influenza A viruses (FLUAV), and porcine epidemic diarrhea virus (PEDv), which are endemic in many high intensity swine production systems and continue to challenge producers and veterinarians (8–11). Recent virus outbreaks such as those caused by ASFV and classical swine fever virus (CSFV) have drastically impacted trade and the overall global swine market (4, 12). Novel swine viruses such as PCV3, swine acute diarrhea syndrome coronavirus (SADS-CoV), may lead to clinical disease, while others such as porcine toroviruses (PToV), porcine bocavirus (PBoV), torque teno sus virus (TTSuV) are mostly subclinical. However, the risk persists for these viruses to cause substantial disruptions to the swine industry. Some viruses detected in pigs may also pose a threat to public health, such as Nipah virus and Hepatitis E virus (HEV) (13, 14).

This Research Topics comprises of six original research articles about characterization of ASFV and FLUAV, vaccines against pseudorabies virus (PRV), inactivation of ASFV, multiplex diagnostics of ASFV, CSFV, and PRRSV, and characterization of porcine parvovirus 7 (PPV7). Two brief research reports are also included about PRV.

## Brief research reports

Tan et al. reported a naturally occurring recombinant event in Hunan province, China between PRV classical strain and its derived HB-98 live attenuated vaccine strain, highlighting safer vaccines and/or vaccination protocols are needed. The isolated HN-2019 the glycoprotein E (gE) and gG genes of the PRV classical strain but the TK gene of the HB-98 vaccine strain. The recombinant strain replicated to similar titers as the parental strains *in vitro* but resulted in intermediary level of replication and disease *in vivo*.

Tan et al. investigated the seroprevalence of PRV in Hunan province in China during 2020 and 2021 and the awareness of the risks of this infectious agent among swine practitioners. In the study, 18,812 pig serum samples and 1,634 tissue samples were tested from 798 farms. The authors found that 8.9% of animals and 20.2% of farms were seropositive for PRV-gE antibodies, and 2.3% of tissues were positive for PRV nucleic acids, demonstrated PRV is prevalent in China. However, workers were mostly unaware of the risks of PRV to humans.

## Original research articles

Nuanualsuwan et al. investigate the thermal inactivation of ASFV in three swill formulae with different nutritive compositions at the temperatures of 60, 70, 75, and 80°C. The authors also defined a prediction model to predict decimal reduction time (DRT or *D*_T_) for other inactivation temperatures. The mean *D*_60_, *D*_70_, *D*_75_, and *D*_80_ of ASFV in three swill formulae were in the ranges 23.21–33.47, 5.83–10.91, 2.15–2.22, and 1.36–1.47 min, respectively. Based on the results, the estimated time for inactivation for the recommended temperatures by the World Organization for Animal Health (WOAH; 90°C) and Food and Agriculture Organization (FAO; 70°C) are 4 and 119 min, respectively.

Shi et al. characterized the ASFV strains circulating in Guangxi province, southern China in the ongoing outbreak. For this study, 336 tissue samples collected from domestic pigs that died with severe hemorrhagic disease in 86 farms during 2019–2020 were tested, and 66 ASFV strains were sequenced and genetically characterized. Phylogenetic analysis showed that genetically diverse strains circulated in the region in 2019–2020, including strains of the genotypes I and II, as well as serogroups 4 and 8, the majority belonging to genotype II and serogroup 8. Interestingly, both wildtype and gene-deleted ASFV strains were identified.

Shi et al. reported the development and testing of a new multiplex crystal digital PCR (dPCR) for differential detection of ASFV, CSFV, and PRRSV, a modified version of the conventional PCR. The assay targets detection of p72, 5′ untranslated region (UTR), and ORF7 genes of each virus, respectively. The assay showed high specificity and sensitivity, capable to detecting low levels of ASFV, CSFV, and PRRSV, but no non-specific detection of other swine viruses was observed. This assay proved to be accurate and a reliable tool for screening for ASFV, CSFV, and PRRSV infection, and could aid in the rapid detection of outbreaks.

Ren et al. compares the efficacy of a commercially available Bartha-K61 vaccine and an rPRV/XJ5-gI–/gE–/TK– prototype vaccine against lethal challenge with a PRV variant strain. Both vaccines were administered the same dose to 12 week-old pigs and resulted in protection, with all vaccinated pigs surviving challenge. Pigs were equally protected against weight loss, pathology, and virus shedding. These results differe from previously published studies when younger pigs were used, demonstrating the two vaccine can be effective in a less PRV susceptible age.

Wen et al. reported the first detection of PPV7 in Inner Mongolia Autonomous Region, China. More than 28% of animals and 60% of herds were positive for PPV7, and co-infection with PCV2 and PCV3 was often observed. The authors sequenced and characterized four strains, demonstrating that all Mongolian PPV7 clustered together, and showed multiple amino acid mutations compared to reference strains.

Osorio-Zambrano et al. characterized the influenza A virus circulating in swine in Colombia before, during, and after the 2009 H1N1 pandemic. Ten virus isolates were selected from a repository, subtyped, and sequenced. Strains isolated in 2008, prior to the pandemic, carried an HA segment from the North American clade 1.A.1 classical lineage. After the introduction of the 2009 H1N1 virus, all isolates contained the HA and NA genes from pandemic clade 1A.3.3.2, demonstrating the pandemic lineage replaced the previously circulating classical H1N1 lineage. Interestingly, evolution of the FLUAV gene segments within the studied strains followed a geographic pattern.

## Author contributions

All authors listed have made a substantial, direct, and intellectual contribution to the work and approved it for publication.

## Conflict of interest

The authors declare that the research was conducted in the absence of any commercial or financial relationships that could be construed as a potential conflict of interest.

## Publisher's note

All claims expressed in this article are solely those of the authors and do not necessarily represent those of their affiliated organizations, or those of the publisher, the editors and the reviewers. Any product that may be evaluated in this article, or claim that may be made by its manufacturer, is not guaranteed or endorsed by the publisher.
